# The Effects of Atorvastatin on Arterial Stiffness in Male Patients with Type 2 Diabetes

**DOI:** 10.1155/2015/846807

**Published:** 2015-04-30

**Authors:** Colin Davenport, David T. Ashley, Eoin P. O'Sullivan, Claire M. McHenry, Amar Agha, Christopher J. Thompson, Donal J. O'Gorman, Diarmuid Smith

**Affiliations:** ^1^Department of Academic Endocrinology, Beaumont Hospital, Dublin, Ireland; ^2^School of Health and Human Performance, Dublin City University, Dublin, Ireland

## Abstract

Statin therapy improves lipid profiles and reduces vascular inflammation, but its effects on central arterial stiffness in type 2 diabetes are unclear. The aim of this study was to determine whether statin therapy reduces central arterial stiffness, in a dose-dependent manner, in male patients with type 2 diabetes. Fifty-one patients ceased statin therapy for 6 weeks, followed by randomisation to either 10 or 80 mg of atorvastatin. At randomization, 3 and 12 months, central arterial stiffness was measured via carotid-femoral pulse wave velocity (PWV), along with serum markers of vascular inflammation including high-sensitivity c-reactive protein (hsCRP) and osteoprotegerin (OPG). PWV decreased from 10.37 ± 1.30 to 9.68 ± 1.19 m/sec (*p* < 0.01 from baseline) at 3 months and 9.10 ± 1.17 m/sec (*p* < 0.001 from baseline) at 12 months. hsCRP and OPG decreased significantly at 3 and 12 months. Reductions in PWV did not differ significantly between the groups. Baseline PWV and OPG values correlated strongly (*r* = 0.48, *p* < 0.01), as did their response to atorvastatin over 12 months (*r* = 0.36 delta-OPG and delta-PWV, *p* < 0.01). Atorvastatin therapy appeared to reduce central arterial stiffness in male type 2 diabetes, with no dose-dependent effect observed. The correlation observed between reductions in PWV and OPG suggests that atorvastatin reduces PWV via direct anti-inflammatory effects on the vasculature.

## 1. Introduction

Type 2 diabetes mellitus is associated with a variety of adverse cardiovascular (CV) features, including increased stiffness of the central arteries when compared to age-matched controls [1, 2]. Elevated central arterial stiffness exerts numerous detrimental effects on the vasculature, including increased systolic blood pressure (BP), left ventricular hypertrophy, and decreased diastolic BP, which in turn is associated with reduced coronary artery perfusion during diastole and decreased perfusing pressures in the peripheral tissues [3]. Central arterial stiffness can be quantified via measurement of carotid-femoral pulse wave velocity (PWV), with elevated PWV indicating elevated stiffness. The importance of central arterial stiffness to CV disease was recently underscored by a meta-analysis of 17 longitudinal studies, in which it was reported that, for each standard deviation (SD) increase in PWV, the age and sex adjusted risk of CV events increased by 47% [4].

The pathophysiology of arterial stiffness is, at present, incompletely understood and appears to be multifactorial, with age, atherosclerotic inflammation, endothelial dysfunction, renal failure, and vascular calcification all capable of contributing to the increased muscle tone and decreased distensibility that characterizes stiffened arteries [5]. As the statin class of medications has been shown to improve atherogenic lipid profiles, along with exerting direct anti-inflammatory effects on the vascular wall, it has been hypothesized that statins may be capable of reducing central arterial stiffness [6]. Preliminary data from euglycemic populations with hypertension or dyslipidemia support this hypothesis [7, 8]. A 2010 systematic review by Rizos et al., however, highlighted the limited evidence base in this area and recommended that additional prospective trials be performed before any conclusions are reached [9]. Furthermore, and with respect to statin therapy and arterial stiffness in type 2 diabetes, it is unclear if data from euglycemic cohorts are applicable to diabetes, as patients with type 2 diabetes typically suffer from multiple promoters of arterial stiffness, including hyperglycemia, vascular calcification, renal dysfunction, and a high prevalence of inflammation, hypertension, and dyslipidemia [2, 3]. Therefore, despite the widespread use of statins in type 2 diabetes, there is insufficient evidence regarding the effects of statins on arterial stiffness in patients with diabetes. Accordingly, the primary aim of this study was to determine whether statin therapy using atorvastatin reduced central arterial stiffness in a type 2 diabetes population, and whether this effect, if present, depended on the dose of atorvastatin. In addition to this aim, we also compared any changes in PWV observed with changes in (a) lipid profiles and (b) biomarkers of vascular inflammation as measured by high-sensitivity c-reactive protein (hsCRP) and osteoprotegerin (OPG) [10, 11]. Statins can affect the arterial wall both indirectly (by inhibiting HMG-CoA reductase activity in the liver and leading to less atherogenic circulating lipid profiles) and directly (by exerting a variety of anti-inflammatory effects on vascular cell populations) [6]. The secondary aim of the research, therefore, was to provide preliminary data on the potential mechanisms whereby statins may affect arterial stiffness.

## 2. Materials and Methods

This was a prospective, open-label trial of atorvastatin, in which male patients with type 2 diabetes with microalbuminuria ceased taking statins for 6 weeks and were subsequently randomized to either 10 or 80 mg of atorvastatin for 12 months. Ethical approval was granted by the hospital's ethics committee and the Irish Medicines Board.

As both arterial stiffness and circulating OPG concentrations may differ between males and females, by design the present study was limited to male patients only [12, 13]. While this approach limited the applicability of the results to males only, it also allowed us to avoid the confounding effects of gender when interpreting our results. Males with documented microalbuminuria (early morning urine albumin-creatinine ratio >2.5 mg/mmol on two separate occasions, 3 months apart, with no other cause identified) were recruited to ensure that all subjects were at high risk for both CV disease and arterial stiffness [14]. Exclusion criteria consisted of malignancy, severe renal impairment (eGFR < 60 ml/min/1.73m^2^), type 1 diabetes, the use of bisphosphonates (potential modifiers of arterial stiffness), and a coronary or cerebrovascular event within the previous 6 months. We also required that all subjects be stable (no change required in the preceding 6 months) on their diabetes and antihypertensive medications prior to entry into the study.

Following recruitment, patients underwent a 6-week washout period during which they stopped taking any statin mediation. Patients then attended the diabetes day centre for a randomization visit, between 8 am and 10 am, having fasted from midnight the night before, during which they underwent measurement of carotid-femoral PWV and provided serum samples, before being randomized, in an open-label fashion, to either 10 or 80 mg of atorvastatin. The standard minimum and maximum dose of atorvastatin prescribed in our country were used in an approach designed to maximize any dose-dependent effects between the two groups [15]. Patients then returned to the centre after 3 and 12 months of treatment, at which time points PWV was reassessed and fasting serum samples were provided once more.

Carotid-femoral PWV was measured using the Vicorder device (Skidmore Medical, Bristol, UK). The same operator took all measurements and was blinded to atorvastatin dose. Size-specific cuffs were placed around the femoral and carotid arteries simultaneously and the distance from the suprasternal notch to the upper limit of the femoral cuff was measured. Measurement was taken under standardised conditions as per guidelines on PWV analysis [16]. Three readings were taken for each patient, and the average of the three was used for analysis. Following PWV testing, blood was drawn, centrifuged, and stored as serum at −80°C for subsequent analysis. HsCRP was measured on the Randox Daytona analyser (Randox, Antrim, Northern Ireland). Serum levels of total OPG were measured using a commercial enzyme-linked immunosorbent assay (ELISA) kit (Biomedica, Vienna, Austria) with intra- and interassay variations of <5% and a minimal detection limit of 0.014 pmol/L. Glycated hemoglobin (HbA1c), triglycerides, and total, low-density lipoprotein (LDL) and high density lipoprotein (HDL) cholesterol were measured using standard laboratory techniques.

Statistical analysis was carried out using SPSS statistical package (version 16.0; SPSS Inc., Chicago, IL, USA). Data are expressed as mean ± standard deviation (SD), or median (25th–75th centile) as appropriate. With regard to whether atorvastatin exerted a dose-dependent effect on PWV and based on previous studies in euglycemic cohorts, we calculated that a sample size of at least 24 in each group was necessary for 80% power to detect a difference of 1 m/sec between the two groups. The effects of atorvastatin on study variables were assessed via one-way ANOVA, with differences between the 10 and 80 mg groups compared using an unpaired *t*-test. Significance was set at *p* < 0.05.

## 3. Results

In total, 55 patients were recruited into the study, with 51 completing the 12-month timeframe (25 in the 10 mg atorvastatin group and 26 in the 80 mg atorvastatin group). Reasons for not completing included noncompliance (*n* = 2) and persistent dyslipidemia requiring additional therapy (*n* = 2). As this study was primarily designed to detect whether atorvastatin exerts an effect on central arterial stiffness, only data from patients who completed the study timeframe were included in the final analysis.

Baseline characteristics of the patients, divided into the 10 and 80 mg groups, are displayed in Table 1. All patients were Caucasian. There were no significant differences between the groups in terms of age, duration of diabetes, BMI, HbA1c, blood pressure, smoking history, renal function, lipid profiles, or the use of diabetes or BP medications. PWV was also comparable between the 10 and 80 mg groups (10.47 ± 1.11 versus 10.28 ± 1.5 m/sec), as were hsCRP (2.88 ± 2.96 versus 2.95 ± 2.48 mg/dL) and OPG (5.44 ± 1.21 versus 5.62 ± 1.58 pmol/L). PWV and OPG demonstrated a strong positive correlation at baseline (*r* = 0.48, *p* < 0.01).

With regards to the effects of atorvastatin on the entire study cohort, PWV decreased from 10.37 ± 1.30 to 9.68 ± 1.19 m/sec after 3 months (*p* < 0.01 from baseline) and 9.10 ± 1.17 m/sec after 12 months (*p* < 0.001 from baseline). hsCRP levels also decreased significantly, from 2.91 ± 2.53 to 1.88 ± 1.64 mg/dL at 3 months (*p* < 0.001 from baseline) and 1.76 ± 1.63 mg/dL after 12 months (*p* < 0.05 from baseline). OPG levels decreased from 5.53 ± 1.39 to 4.81 ± 1.30 pmol/L after 3 months (*p* < 0.01 from baseline) and 4.65 ± 1.25 pmol/L after 12 months (*p* < 0.05 from baseline). Total cholesterol decreased from 5.58 ± 1.08 to 3.79 ± 0.98 mmol/L after 3 months (*p* < 0.05 from baseline) and 3.60 ± 0.99 mmol/L after 12 months (*p* < 0.001 from baseline), while LDL decreased from 3.46 ± 0.70 to 1.92 ± 0.67 mmol/L after 3 months (*p* < 0.01 from baseline) and 1.61 ± 0.57 mmol/L after 12 months (*p* < 0.001 from baseline).

**Figure 1:**
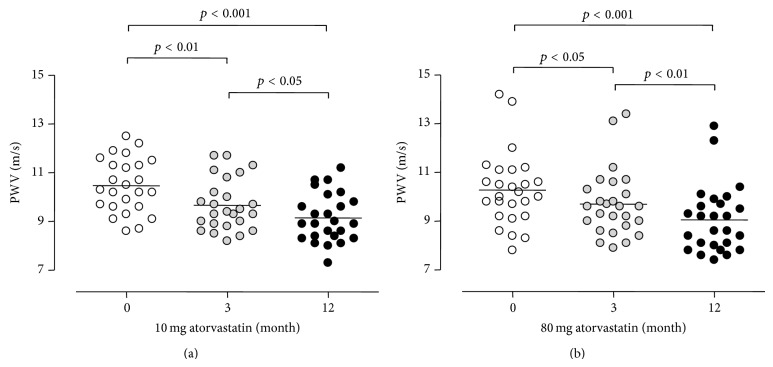
Reduction in PWV in 10 (a) and 80 mg (b) groups, at 3 and 12 months. PWV: pulse wave velocity.

**Figure 2:**
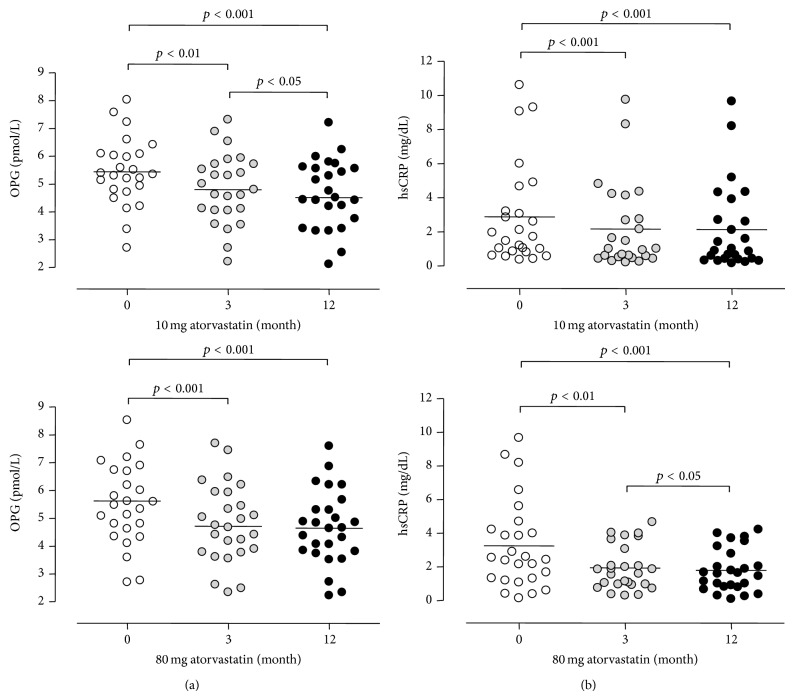
Reduction in OPG (a) and hsCRP (b) in 10 and 80 mg groups at 3 and 12 months. OPG: osteoprotegerin; hsCRP: high-sensitivity c-reactive protein.

**Figure 3:**
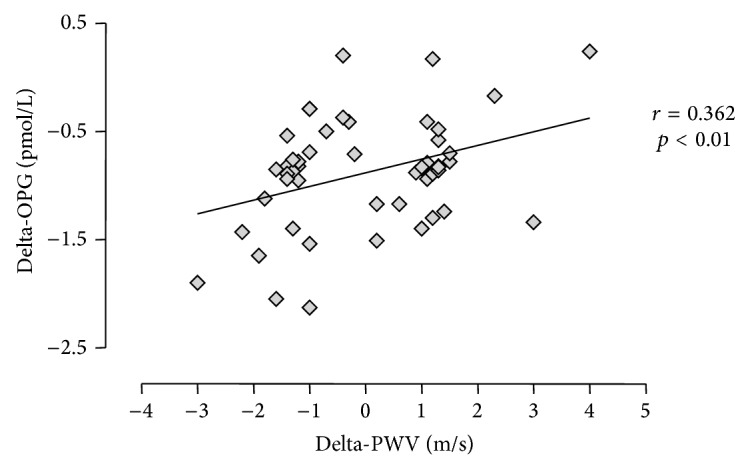
Correlation between change in PWV and change in OPG from baseline to 12 months in each patient. OPG: osteoprotegerin; PWV: pulse wave velocity.

When the 10 and 80 mg atorvastatin groups were compared (Figures 1 and 2), there was no evidence that the dose of atorvastatin affected the reductions in PWV, hsCRP, or OPG observed. In contrast to PWV and OPG, a dose-dependent effect was observed between atorvastatin dose and the reduction in total cholesterol and LDL, with significantly greater reductions noted in the 80 mg group for both total cholesterol and LDL (*p* < 0.001 for a difference between the response of both variables between the 10 and 80 mg groups). The changes in PWV were noted to correlate positively with the changes in OPG over the 12 months (*r* = 0.36 delta-OPG and delta-PWV, *p* < 0.01), as demonstrated in Figure 3, but not with the change in hsCRP, total cholesterol, or LDL. There were no significant changes in BMI, HbA1c, or BP over the course of the study.

## 4. Discussion

The primary finding of this study was that PWV decreased significantly following the commencement of atorvastatin, suggesting that statins may exert additional benefits on CV disease in type 2 diabetes patients with microalbuminuria by reducing central arterial stiffness. Only two studies have previously performed prospective research on statins and PWV in type 2 diabetes. The first, by Mukherjee et al., reported that atorvastatin improved arterial stiffness in 71 Indian patients with type 2 diabetes [17]. Notably, this was a low-risk cohort (normotensive, normal lipid profiles) whose arterial stiffness was assessed via brachial-ankle PWV and not the gold standard of carotid-femoral PWV. The second study, by Shinohara et al., examined the effects of atorvastatin in a Japanese population and reported a trend towards an improvement in central arterial stiffness [18]. Once again, this was a low-risk type 2 cohort and one which did not follow current guidelines on the measurement of PWV. As such, it was unclear if the data from these studies were applicable to the general type 2 diabetes population. With regard to retrospective studies a secondary analysis of the ADDITION-Denmark study indicated that carotid-femoral PWV decreased in patients with type 2 diabetes randomized to intensive risk factor management when compared to conventional care but statin use was similar between the two groups in this study (80% versus 72%), and so it was not possible to determine if statins accounted for the differences in PWV [19]. Ultimately, by demonstrating a beneficial effect from atorvastatin on PWV in the presence of multiple risk factors for arterial stiffness, our study expands the limited evidence base in this area to include males with type 2 diabetes and microalbuminuria and indicates the benefits of statin in this high CV-risk group.

The secondary findings of this study were that atorvastatin reduced PWV along with hsCRP and OPG by the same amount regardless of whether patients received the 10 or 80 mg dose and that the reduction in PWV observed correlated with the reduction in OPG, but not with the reductions in hsCRP, total cholesterol, or LDL. With regard to the discrepancy observed between the two biomarkers of vascular inflammation, it should be noted that the high degree of intrasubject variation in hsCRP levels diminished the statistical significance of findings related to this biomarker of inflammation, whereas OPG showed lower intrasubject variability when compared to hsCRP. Furthermore, we note that OPG may exhibit a closer relationship with vascular inflammation than hsCRP, with the former biomarker produced directly by vascular cells and the latter produced in the liver in response to varied inflammatory stimuli. In combination, these preliminary data suggest that atorvastatin may reduce PWV via direct anti-inflammatory effects, as opposed to the lipid-lowering effects of statins. Interestingly, the possibility of a direct effect of statins on arterial stiffness has previously been raised by Orr et al. who noted that, by decreasing inflammation and oxidative stress within the vasculature, statins could, theoretically, increase nitrous oxide bioavailability, leading to diminished smooth muscle tone and improved arterial wall distensibility [7]. It is important to note, however, that the correlations observed in the present study are hypothesis-generating only, and additional research will be necessary to examine how statins affect CV hemodynamics.

The strengths of the present study include its use of carotid-femoral PWV, the gold-standard technique for the measurement of central arterial stiffness and one which exhibits a clear relationship with CV event rates in multiple patient populations. The use of male patients with microalbuminuria allowed us to demonstrate the ability of atorvastatin to affect arterial stiffness despite the presence of multiple risk factors for stiffness in a high CV-risk cohort. Additional research will be necessary to test these effects in a female population. With regard to study design, we note that while an optimal assessment of medication effects on a patient population would include a placebo arm, we felt that the high CV-risk status of the majority of patients with T2DM and especially those with microalbuminuria precluded this approach in the present study on ethical grounds.

In conclusion, our preliminary data demonstrate, for the first time, the ability of atorvastatin, at low or high dose, to improve central arterial stiffness in male Caucasian patients with type 2 diabetes. This effect appeared to be mediated through direct anti-inflammatory as opposed to lipid-lowering effects. Additional research is merited to determine if statins may have a role as an agent with which to improve CV hemodynamics in patients with type 2 diabetes.

## Figures and Tables

**Table 1:** Baseline characteristics of the study cohort, divided into 10 and 80 mg groups.

	10 mg	80 mg	*p* value
	(*n* = 25)	(*n* = 26)	
Age (years)	67 (46–84)	67 (43–85)	0.72
Total cholesterol (mmol/L)	5.5 ± 1	5.6 ± 1.2	0.75
Low-density lipoprotein (mmol/L)	3.6 ± 0.6	3.4 ± 0.8	0.41
HbA1c (mmol/mol)	52.5 ± 4.1	51.2 ± 4.5	0.39
HbA1c (%)	6.9 ± 0.8	6.8 ± 0.8	0.39
eGFR (mL/min/1.73 m^2^)	81.7 (61.7–141.4)	98.7 (60.5–145.5)	0.67
Smoking (%)	12 (3/25)	27 (7/26)	0.29
Duration of diabetes (years)	7 (1–22)	8 (2–19)	0.78
Body mass index (kg/m^2^)	31.2 ± 6.7	32.4 ± 4.7	0.85
Systolic BP (mmHg)	140 (120–157)	139 (130–150)	0.87
Diastolic BP (mmHg)	80 (72–90)	80 (59–90)	0.89

Data are presented as mean ± standard deviation (SD), median (25th–75th centile), or absolute numbers as appropriate. BP: blood pressure; eGFR: estimated glomerular filtration rate; HbA1c: glycated hemoglobin.
